# Systematic expression analysis of the CELSR family reveals the importance of CELSR3 in human lung adenocarcinoma

**DOI:** 10.1111/jcmm.16497

**Published:** 2021-04-03

**Authors:** Yishuai Li, Longyu Zhu, Ran Hao, Yuejun Li, Qinfei Zhao, Shujun Li

**Affiliations:** ^1^ Department of Thoracic Surgery The Second Hospital of Hebei Medical University Shijiazhuang China; ^2^ Department of Thoracic Surgery Hebei Chest Hospital Shijiazhuang China; ^3^ Department of Radiotherapy The Fourth Hospital of Hebei Medical University Shijiazhuang China; ^4^ School of Nursing Hebei Medical University Shijiazhuang China; ^5^ Department of Oncology The Third Affiliated Hospital of Hunan University of Chinese Medicine Zhuzhou China; ^6^ Department of Oncology The First Affiliated Hospital of Hunan College of Traditional Chinese Medicine Zhuzhou China; ^7^ Department of Laboratory Medicine First Affiliated Hospital of Gannan Medical University Ganzhou China

**Keywords:** CELSR3, chemokines, functional network analyses, immune infiltrates, LUAD, prognosis

## Abstract

Cadherin EGF LAG seven‐pass G‐type receptors (CELSRs) are involved in the progression of various types of cancer. *CELSR3*, a crucial signalling molecule in the WNT/PCP pathway, is believed to be associated with tumorigenesis and metastasis. However, its role in lung adenocarcinoma (LUAD) remains unclear. In this paper, we analysed the expression of CELSR family members using the Oncomine, GEPIA and UALCAN databases. We used a Kaplan‐Meier plotter to assess the effect of CELSRs on tumour prognosis. Next, gene ontology (GO), KEGG pathway, miRNA target, kinase target and transcription factor‐target enrichment were analysed by GSEA. Simultaneously, we conducted functional assays including cell viability, colony formation and transwell assays, to determine the oncogenic role of *CELSR3* in LUAD. Finally, we used the TIMER and TISIDB databases to analyse the correlation between *CELSR3* and immune infiltration and the potential chemokine receptor axis causing immune cell expression. High expression of *CELSR3* is in LUAD predicts poor prognosis and early progression of the tumour. KEGG and GO enrichment analysis revealed the functional relationship between *CELSR3* and cell adhesion, the cell cycle, and DNA replication. Down‐regulation of *CELSR3* suppressed cell proliferation to a significant extent, in addition to inhibiting invasion and migration in LUAD cells. Finally, *CELSR3* expression was significantly correlated with the infiltration level of CD8+T cells through the CCL17/CCR4 axis in LUAD. These results indicate that *CELSR3* can serve as a prognostic biomarker for determining prognosis and immune infiltration in LUAD.

## 1 INTRODUCTION

Globally, lung cancer is one of the leading causes of cancer‐related deaths, with non‐small‐cell lung cancer (NSCLC) accounting for approximately 85%.^1^ Lung cancer is classified in different cancer types, and lung adenocarcinoma (LUAD) is the major histological type of lung cancer.^2^, ^3^ Despite recent progress in clinical surgical technique^4^ and comprehensive therapy,^5^ the prognosis for LUAD remains poor because of treatment resistance, local recurrence^6^ and distant metastasis.^7^ Thus, there is an urgent need to both identify LUAD biomarkers for early specific diagnosis and prognosis evaluation, and further develop strategies to treat this fatal disease.^8^

Cadherin EGF LAG seven‐pass G‐type receptors belong to the flamingo subfamily of the cadherin superfamily. CELSRs are known to be involved in the regulation of contact‐dependent neurite growth^9^ and may play an important role in the development and metastasis of different cancers.^10^ The Wnt signalling pathway is thought to play a key role in tumorigenesis through the typical Wnt/catenin cascade. However, a growing body of evidence has revealed the previously unrecognized role of non‐canonical Wnt/planar cell polarity (PCP)^11^ signalling in cancer progression, metastasis, evasion and angiogenesis. Thus, *CELSR3*, as a crucial signalling molecule in the WNT/PCP pathway, is believed to be associated with tumorigenesis and metastasis.

However, the role of *CELSR3* in the LUAD development remains unknown, as does whether *CELSR3* is a specific marker in LUAD. In this study, we used the Oncomine, GEPIA and UALCAN public databases to analyse the CELSR family expression. Then, we used the Kaplan‐Meier(KM) Plotter to evaluate the association between CELSR family mRNA levels and disease prognosis in patients with LUAD. In addition, we explored the relationship between *CELSR3* mRNA expression levels and clinical subgroups and tumour‐infiltrating immune cells in patients with LUAD.

## 2 MATERIALS AND METHODS

### 2.1 Oncomine database analysis

Oncomine database is an online cancer microarray database that aimed at facilitating discovery from genome‐wide expression analyses (https://www.oncomine.org/resource/login.html).^12^, ^13^ The expression levels of CELSR mRNA (log2‐transformed) in lung cancer tissues and normal tissues were searched using Oncomine database, and the transcription levels of CELSRs in all lung cancer tissues were determined for statistical comparison. To obtain the important CELSR probes, we set the following thresholds: *P*‐value < .05, fold change >1.5 and gene ranks in the top 10%.

### 2.2 UALCAN analysis

UALCAN is an interactive web resource that provides comprehensive cancer transcriptome date for in‐depth analysis of The Cancer Genome Atlas (TCGA) gene expression and clinical data from 31 types of cancer (http://ualcan.path.uab.edu ).^14^, ^15^ Using UALCAN, we also analysed the relative *CELSR3* expression in LUAD across tumours and corresponding normal tissues, in addition to different tumour subgroups.

### 2.3 GEPIA analysis

The GEPIA (http://gepia.cancer‐pku.cn/index.html)^16^ is an interactive website based on TCGA and GTEx‐based project, where RNA sequencing expression in tumours and corresponding normal tissues can be analysed. We used GEPIA to analyse the correlation of TCGA expression data and the Spearman method to determine the correlation coefficient between two genes.

### 2.4 KM Plotter survival analysis

The prognostic value of the *CELSRs* mRNA expression was assessed by the Kaplan‐Meier plotter (www.kmplot.com),^17^ an online database containing gene expression data and survival information for lung cancer patients. Using this database, we analysed the overall survival (OS) and first progression (FP) of patients with LUAD. The patient samples were divided at the median expression into two groups with either high expression or low expression, and evaluated using the Kaplan‐Meier survival plot [*P*‐value < .05, false detection rate (FDR) < 0.05). The risk ratio (HR) had a 95% confidence interval and log‐rank *P*‐value.

### 2.5 LinkedOmics analysis

The LinkedOmics database is a website used to analyse 32 TCGA cancer‐related datasets (http://www.linkedomics.org/login.php).^18^ We used the LinkFinder module of LinkedOmics to study the differentially expressed genes related to *CELSR3* in the TCGA LUAD (n = 515). Pearson correlation coefficient was used for statistical analysis of the results, and LinkFinder was used to establish a statistical diagram of a single gene. Functional enrichment analysis was applied using the Web‐based Gene SeT Analysis Toolkit (WebGestalt).^19^ The data in the LinkFinder results were signed and sequenced, and we used gene set enrichment analysis (GSEA) for gene ontology (GO) analysis, KEGG and network analysis. We used the Molecular Signatures Database (MSigDB)^20^ to analyse the network (FDR <0.05; and 500 simulations were performed).

### 2.6 Tumour immune estimation resource (TIMER) database analysis

Tumour immune estimation resource is a comprehensive resource for the systematic analysis of immune infiltrates across 32 tumour types (https://cistrome.shinyapps.io/timer/).^21^ TIMER employs a previously published statistical method^22^ to infer the abundance of tumour‐infiltrating immune cells (TIICs) from gene expression profiles. We analysed the expression of *CELSR3* in different types of lung cancer and the correlation of *CELSR3* expression with infiltration of immune cell, including B cells, CD8+ T cells, CD4+ T cells, macrophages, neutrophils and dendritic cells (DCs), through gene patterns.^23^ Moreover, we studied the correlations between *CELSR3* expression and genetic markers in TIICs through related modules.

### 2.7 TISIDB analysis

The TISIDB is a web portal (http://cis.hku.hk/TISIDB) that combines 4176 records from 2530 publications and reports on 988 genes associated with anti‐tumour immunity. We can explore the function of genes of interest and their role in tumour immune interactions by analysing the TISIDB database and through high‐throughput data analysis and literature mining.^24^ On the X‐axis, *CELSR3* is used as the gene symbol, and on the Y‐axis, the TIICs‐related gene marker is used as the gene symbol. We used Log2 RSEM to determine the gene expression level.

### 2.8 Cell culture and Small Interfering RNA Transfection

We obtained the LUAD A549 and H1975 cell lines from the Cell Bank of Shanghai Biology Institute (Shanghai, China). We cultured these cells in RPMI‐1640 medium supplemented with 10% calf serum (Invitrogen, USA), 100 IU/mL penicillin and 100 IU/mL streptomycin at 37℃ in a humidified atmosphere with 5% CO_2_. For transfection, we cultured the cells to 70% confluence and transfected them with siRNA using Lipofectamine2000 (Invitrogen, CA, USA) following the manufacturer's recommended protocol. We trained the transfected cells in fresh medium for 24 hours under normal conditions and collected them for subsequent analysis. The siRNA sequences for *CELSR3* (SI) and negative control (NC) synthesized by GenePharma (Shanghai, China) are shown in Table S4. The si‐CELSR3#1 sequences were used in cell transfection. The primers used in qRT‐PCR are listed in Table S5.

### 2.9 Cell proliferation assays

#### 2.9.1 Cell counting kit‐8 assay

We transfected A549 and H1975 cells were with either CELSR3 siRNA or an siRNA control (GenePharma, Shanghai, China). Following transfection for 24 hours, the cells were seeded into a 96‐well plate at 5.0 × 10^3^ cells/mL and continuously cultured for 24, 48, and 72 hours. At each time‐point, we added CCK8 reagent (DojinDO, Shanghai, China) to each well and incubated for 2 hours at 37°C. We measured spectrometric absorbance at a wavelength of 450 nm on a microplate reader (Spectra Max M5, MD, USA). Each sample was tested in triplicate, and all experiments were performed three times.

#### 2.9.2 Colony formation assays

We transfected A549 and H1975 cells with either *CELSR3* siRNA or an siRNA control (GenePharma, Shanghai, China). After 24 hours transfection, we seeded 2.5 × 10^3^ A549 cells and 5 × 10^3^ H1975 cells into a six‐well plate. To achieve colony formation, we grew the cells for 6 days in normal culture medium. We stained the colonies with crystal violet.

### 2.10 Transwell assay

We used a Boyden chamber assay to study cell invasion capability. Cells were transfected with either CELSR3 siRNA or an siRNA control (Ruibobio, Shanghai, China). After 24 hours, we trypsinized and resuspended the transfected cells and placed 5.0 × 10^4^ cells in 200 μL RPMI‐1640 medium into the upper chambers (8‐mm pore size; Millipore). We filed the lower chambers with 600 μL complete medium containing 10% FBS. Following 24‐hour incubation of the cells at 37°C, we used a cotton swab to remove non‐invading cells from the top of the chamber. The invading cells on the lower surface of the inserts were fixed and stained with 0.1% crystal violet, and five random fields for each insert were counted at 100× magnification.

### 2.11 Statistical analysis

All statistical analyses were performed using online resources. Survival curves were generated using the Kaplan‐Meier method. The results generated in Oncomine are displayed with Student's *t* test to compare mRNA expression. Kaplan‐Meier plots are shown with a hazard ratio with 95% confidence intervals and log‐rank *P*‐value. GEPIA variance analysis was tested using one‐way ANOVA by defining the disease status (tumour or normal) as variable. All data are presented as the mean ± standard derivation (SD). SPSS 19.0 software (SPSS, Chicago, IL, USA) was applied for the statistical analyses. Spearman's correlation was used to evaluate the correlation of gene expression, and all *P*‐values < .05 were considered statistically significant.

## 3 RESULTS

### 3.1 Up‐regulation of *CELSR3* mRNA expression in human LUAD

**FIGURE 1:**
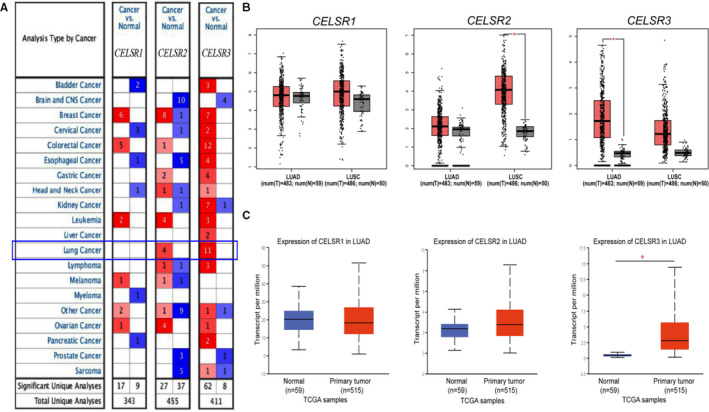
High transcription of *CELSR3* in LUAD. Transcription of CELSR family in Oncomine database (A), GEPIA database (B) and UALCAN database (C)

**FIGURE 2:**
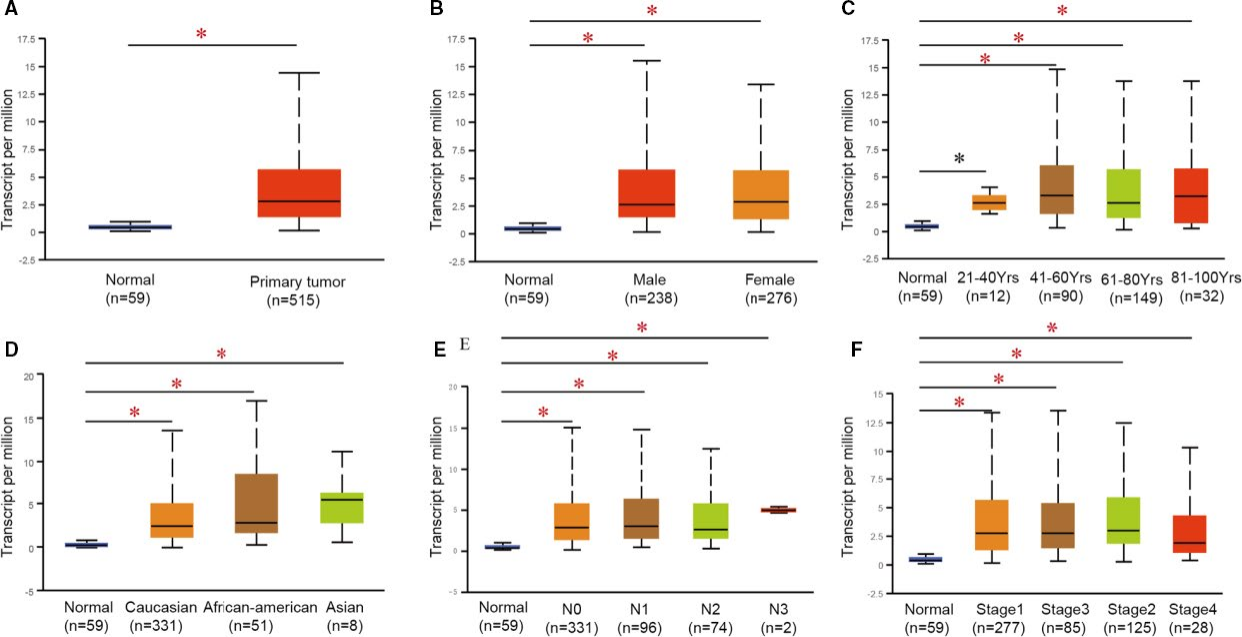
*CELSR3* transcription in subgroups of patients with LUAD, stratified based on sex, age and other criteria (UALCAN). A, Boxplot showing relative expression of *CELSR3* in normal and tumour samples. B, Boxplot showing relative expression of *CELSR3* in normal individuals of either gender or male or female LUAD patients. C, Boxplot showing relative expression of *CELSR3* in normal individuals of any age or in LUAD patients aged 21‐40, 41‐60, 61‐80, or 81‐100 y. D, Boxplot showing relative expression of *CELSR3* in normal individuals of any ethnicity or in LUAD patients of Caucasian, African‐American or Asian ethnicity. E, Boxplot showing relative expression of *CELSR3* in normal individuals or in LUAD patients in grade 1, 2, 3 or 4. F, Boxplot showing relative expression of *CELSR3* in normal individuals or LUAD patients with stage 1, 2, 3 or 4 tumours. Data are mean ± SE. *P < .05

Three CELSR family members have been identified in human cancers. We analysed the transcriptional levels of the CELSRs using the Oncomine database in different tumours and normal tissues of multiple cancer types. Our findings showed that the expressions of *CELSR3* and *CELSR2* were higher in lung tumours tissues compared with the corresponding normal tissues (Figure 1A). Further Oncomine analysis showed that many studies suggested high *CELSR3* expression in LUAD (Table 1). Then, we mined the GEPIA and UALCAN databases, and found that *CELSR3* was the only higher CELSR family member relative to normal tissues in LUAD tissues (Figure 1B,C). Multiple subgroup analysis of clinical and pathological features of 515 LUAD samples from TCGA showed high transcription of *CELSR3*. Moreover, in subgroup analyses based on sex, age, ethnicity, stage and tumour grade, the *CELSR3* transcription was significantly higher in LUAD patients than in healthy individuals (Figure 2).

**TABLE 1:** The significant changes of *CELSR* expression in transcription level between different types of lung cancer and normal lung tissues (Oncomine database)

	Source and/or Reference	Type of Breast Cancer versus Normal Breast Tissue	N	*P‐*Value	Fold chang
CELSR3	Okayama Lung	(Adenocarcinoma vs. Normal)	246	1.09E−18	2.106
	Bhattacharjee Lung	(Adenocarcinoma vs. Normal)	203	0.022	1.664
	Selamat Lung	(Adenocarcinoma vs. Normal)	116	2.19E−17	2.943
	Hou Lung	(Adenocarcinoma vs. Normal)	156	2.77E−10	1.776
	Su Lung	(Adenocarcinoma vs. Normal)	66	2.59E−06	1.72
	Stearman Lung	(Adenocarcinoma vs. Normal)	39	9.20E−06	1.79
	Garber Lung	(Adenocarcinoma vs. Normal)	73	9.60E−04	1.576
	Landi Lung	(Adenocarcinoma vs. Normal)	107	1.00E−11	1.303
CELSR2	Garber Lung	(Squamous carcinoma vs. Normal)	73	7.39E−06	2.4
	Garber Lung	(Adenocarcinoma vs. Normal)	73	1.33E−04	1.599
	Bhattacharjee Lung	(Squamous carcinoma vs. Normal)	203	3.53E−05	3.782
	Hou Lung	(Squamous carcinoma vs. Normal)	156	2.90E−09	2.074

### 3.2 High *CELSR3* expression predicts poor prognosis in LUAD

**FIGURE 3:**
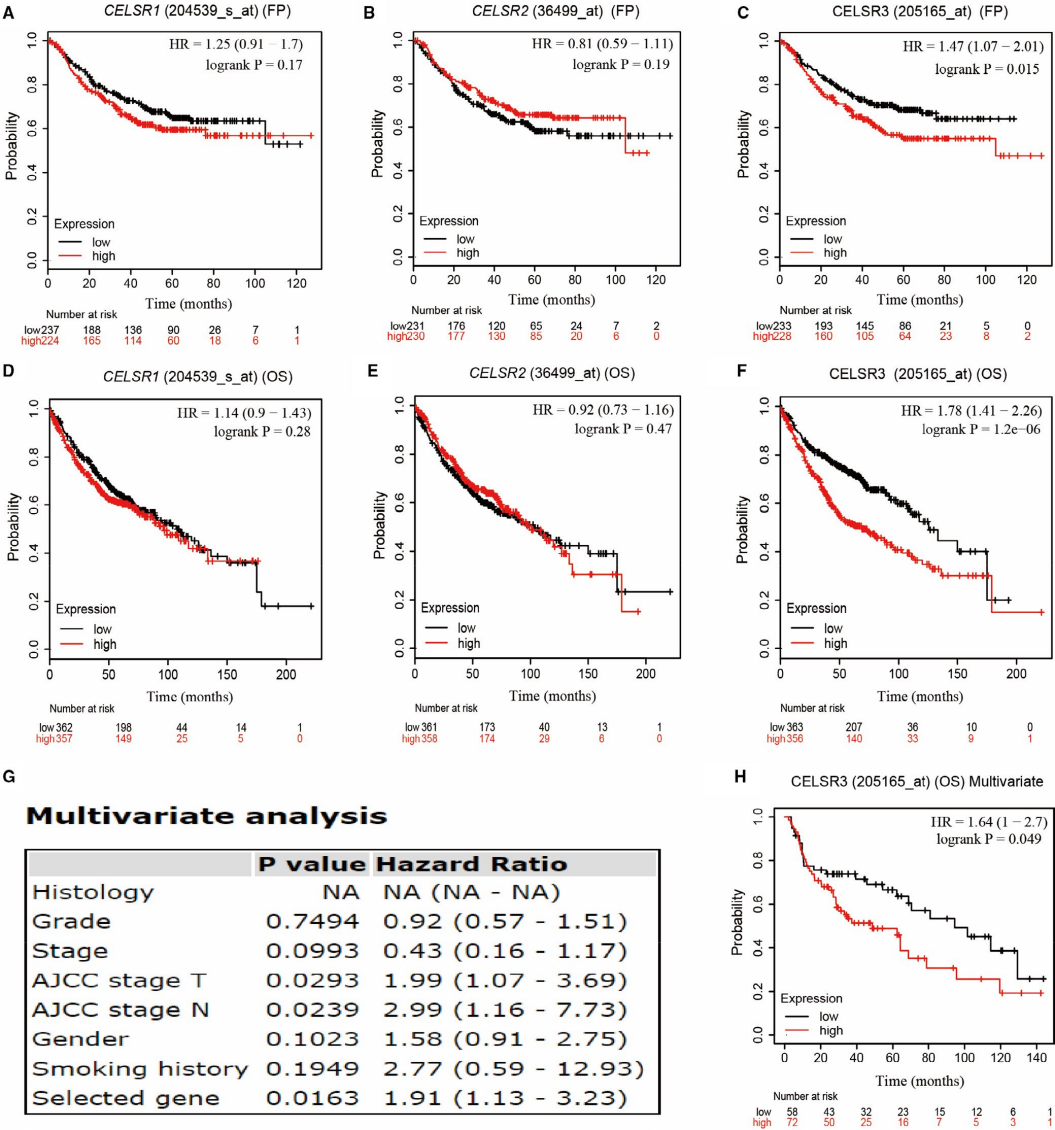
The prognostic value of mRNA level of *CELSR* family in LUAD patients (Kaplan‐Meier plotter)

We used the KM plotter to analyse the correlation between the mRNA levels of *CELSRs* and the prognostic value of patients with LUAD (2015 http://kmplot.com/). The KM curve and log‐rank test analysis revealed a significant association between elevated *CELSR3* mRNA levels and both OS and progression‐free survival (FP) (*P* < .05) (Figure 3) in patients with LUAD. Furthermore, patients with LUAD showing high *CELSR3* expression compared with the median level were predicted to have poor prognosis and early tumour progression. Multivariate analysis showed that the *CELSR3* remained an independent prognostic risk factor for OS of patients with LUAD (*P* < .05). Thus, the *CELSR3* expression can be used as a biomarker for diagnosis, an indicator of treatment viability, and as an indicator of prognostic indicator in LUAD.

### 3.3 GO and KEGG pathway analyses of co‐expression genes correlated with *CELSR3* in LUAD

**FIGURE 4:**
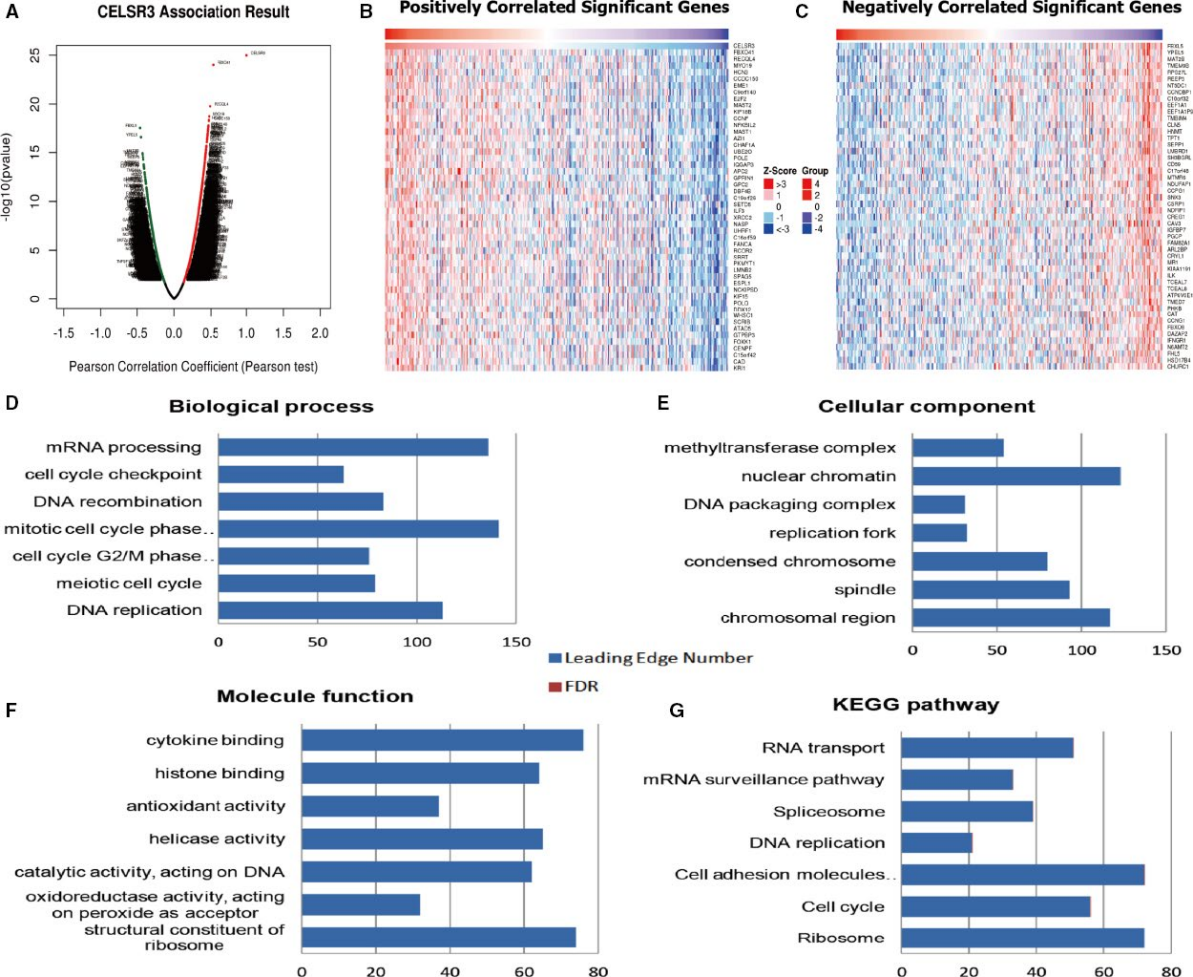
Genes differentially expressed in correlation with *CELSR3* in human lung adenocarcinoma (LinkedOmics). A, A Pearson test was used to analyse correlations between *CELSR3* and genes differentially expressed in LUAD. B, C, Heat maps showing genes positively and negatively correlated with *CELSR3* in LUAD (TOP 50). Red indicates positively correlated genes, and green indicates negatively correlated genes. The significantly enriched GO annotations and KEGG pathways of *CELSR3* co‐expression genes in LUAD were analysed using GSEA. D, Biological processes. E, Cellular components. F, Molecular functions. G, KEGG pathway analysis. The blue column represents the LeadingEdgeNum, and the orange represents the false discovery rate (FDR). The FDR from GSEA in the figure is 0

We analysed the mRNA sequencing data of 515 patients with LUAD in TCGA were analysed using the LinkedOmics functional module. The volcanic diagram (Figure 4A) showed that 2832 genes (dark red dots) were significantly positively correlated with CELSR3, and 2275 genes (dark green dots) were significantly negatively correlated with CELSR3 (false detection rate [FDR] < 0.05). The heat map shows 50 significant gene sets that are positively and negatively correlated with *CELSR3* (Figure 4B,C). GO analysis by GSEA showed that the differentially expressed genes related to *CELSR3* were mainly located in the nuclear chromatin, chromosomal region, spindle, condensed chromosome and replication fork. They are mainly involved in cell cycle checkpoint, G2/M‐phase transition, DNA replication, mitotic cell cycle, cell adhesion molecules, mRNA surveillance pathways and RNA transport. Furthermore, the differentially expressed genes primarily act as structural constituents in cytokine binding, antioxidant activity, histone binding, catalytic activity acting on DNA, and as structural constituents in ribosome and helicase activity (Figure 4D‐F). In agreement, KEGG analysis supported this, showing enrichment of these genes in the cell cycle checkpoint, G2/M‐phase, DNA replication, mitotic cell cycle, cell adhesion molecules, mRNA surveillance pathways and RNA transport (Figure 4G).

### 3.4 *CELSR3* networks of kinase, miRNA and transcription factor targets in LUAD

Gene expression is tightly regulated by multiple layers of mechanisms, including kinase, miRNA and transcription factor (TF) binding. Using GSEA, we studied the kinases, miRNAs and transcription factor‐target networks of *CELSR3* related gene sets to explore the mechanism of action of *CELSR3*. In general, the seven most significant target networks were kinase‐target networks related mainly and in a subtle fashion to the kinases ATR, ATM/PLK1, CDK1, CDK2, CHEK1 and CHEK2 (Table 2). In addition, the miRNA‐target network was associated with MIR‐517, MIR‐503, MIR‐370, MIR‐331, MIR‐197, MIR‐125 and MIR‐412. Finally, the E2F family of transcription factor were the mainly transcription factor‐target network, including E2F1DP1RB_01, E2F1DP1_01, E2F1DP2_01, V$E2F4DP2_01, and V$E2F4DP1_01.

**TABLE 2:** The Kinase, miRNA and transcription factor‐target networks of *CELSR3* in human lung adenocarcinoma (LinkedOmics)

Enriched category	Gene Set	Leading edge number	FDR
miRNA target	TGCACGA,MIR‐517A,MIR‐517C	11	0.002127
	CGCTGCT,MIR‐503	9	0.003191
	CAGCAGG,MIR‐370	53	0.004254
	CCAGGGG,MIR‐331	31	0.009572
	GTGGTGA,MIR‐197	23	0.026802
	CTCAGGG,MIR‐125B,MIR‐125A	91	0.027298
	GGTGAAG,MIR‐412	12	0.040719
Transcription factor target	V$E2F1_Q6	87	0
	V$E2F1DP1_01	90	0
	V$E2F1DP2_01	90	0
	V$E2F4DP2_01	90	0
	V$E2F4DP1_01	89	0
	V$E2F1DP1RB_01	78	0
	V$E2F1_Q6_01	78	0
Kinase target	Kinase_ATR	27	0
	Kinase_CDK2	111	0
	Kinase_CDK1	108	0
	Kinase_CHEK1	69	0
	Kinase_ATM	53	0
	Kinase_PLK1	37	0
	Kinase_CHEK2	14	0

Abbreviations: FDR, false discovery rate from Benjamini and Hochberg from gene set enrichment analysis (GSEA); LeadingEdgeNum, the number of leading edge genes; V$, the annotation found in Molecular Signatures Database (MSigDB) for transcription factors (TF).

### 3.5 Silencing of CELSR3 in LUAD cells inhibits cell proliferation and motility

To explore the relationship between *CELSR3* and **proliferation, cell invasion, and migration of LUAD cells**, we initially focused on the correlations between *CELSR3* and transcription factors/kinases. Our findings showed that *ATR, ATM, PLK1, CDK1, CDK2, CHEK1, CHEK2, E2F1* and *E2F4* were significantly associated with *CELSR3* expression in LUAD (*P* < .01) in the GEPIA databases. Moreover, on the KM plotter website, we found that high expression of *ATR, ATM, PLK1, CDK1, CDK2, CHEK1, CHEK2, E2F1* and *E2F4* predicts poor prognosis in LUAD (Figure S1).

#### 3.5.1 *CELSR3* overexpression can be suppressed by RNA interference

To reveal how *CELSR*3 affects LUAD progression, we conducted an experimental analysis using the human LUAD cell lines A549 and H1975. We used qRT‐PCR to confirm *CELSR3* mRNA expression in LUAD cell lines. Through qRT‐PCR, we found that all siRNA sequences could markedly restrain the mRNA expression of *CELSR3* in A549 and H1975 cells. Moreover, compared with si‐CELSR3#2 and si‐CELSR3#3, si‐CELSR3#1 showed better ability of silencing. Hence, in the subsequent analysis, we used si‐CELSR3#1 for specific RNA interference (Figure S4).

#### 3.5.2 *CELSR3* knockdown inhibits proliferation and ablation of *CELSR3* suppresses migration and invasion in LUAD cell

**FIGURE 5:**
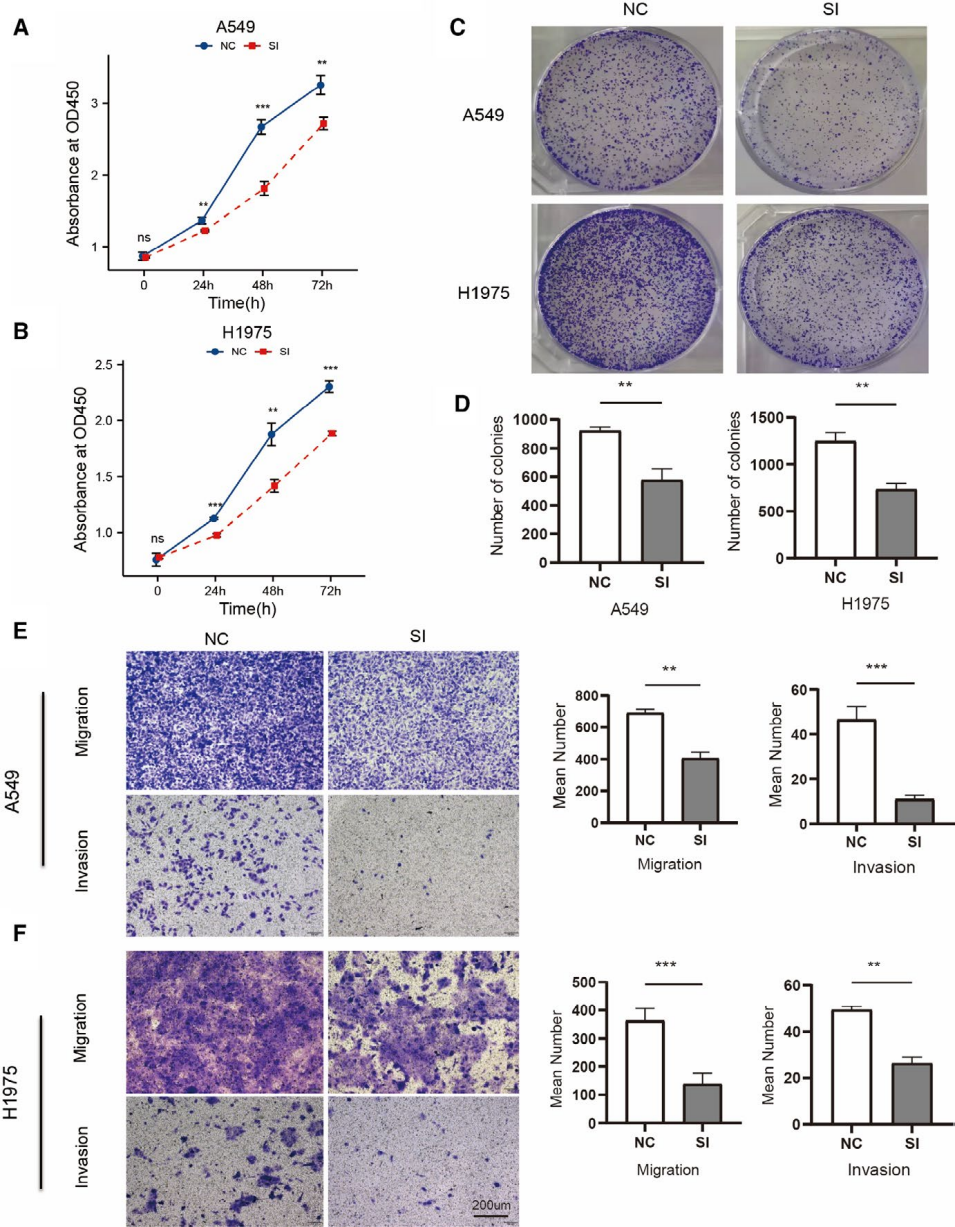
Silencing of *CELSR3* in LUAD cells inhibits cell proliferation and motility. A, B, Silencing of *CELSR3* significantly inhibited cell viability of the H1975 SI and A549 SI cells. C, D, Proliferation in A549 and H1975 cells transfected with NC or si‐*CELSR3* was assessed by the colony‐forming assay (left) and further quantified in the number of colonies of triplicate experiments (right). E, F, Cell migration was assessed by Transwell assay in H1975 and A549 cells after silenced of *CELSR3* for 24 h. The cells migrated into the lower chamber were stained. Cell invasion was evaluated by Transwell assay with Matrigel in H1975 and A549 cells after silenced of *CELSR3* for 24 h (by *t* test analysis, ***P < .001). SI, siCELSR3 cells; NC, negative control cells

We used a CCK8 assay to study how *CELSR3* expression influences LUAD cell proliferation. We performed knockdown of *CELSR3* in the lung cancer cell lines A549 and H1975. The results suggested that *CELSR3* significantly affected cell proliferation (Figure 5A‐B). As shown in Figure 5A, the cell number increased in all groups over time, and *CELSR3* knockdown obviously suppressed the proliferation of A549 and H1975 cells at time‐points 24, 48, and 72 hours, compared with the NC group. This finding was further substantiated by the subsequent colony formation experiment. Moreover, compared with the NC group, there was a significant reduction in the clone number of the si‐CELSR3 group (*P* < .01; Figure 5C,D), suggesting that silencing *CELSR3* significantly reduced the rate of colony formation in LUAD cells. We conducted transwell migration and invasion experiments to ascertain how down‐regulation of *CELSR3* affects A549 and H1975 cell motility. The number of invasive and migrant cells in the si‐CELSR3 group was visibly less than that in NC group (Figure 5E‐F, *P* < .01).

### 3.6 Correlation of *CELSR3* expression with infiltrating CD8+ T cells

**FIGURE 6:**
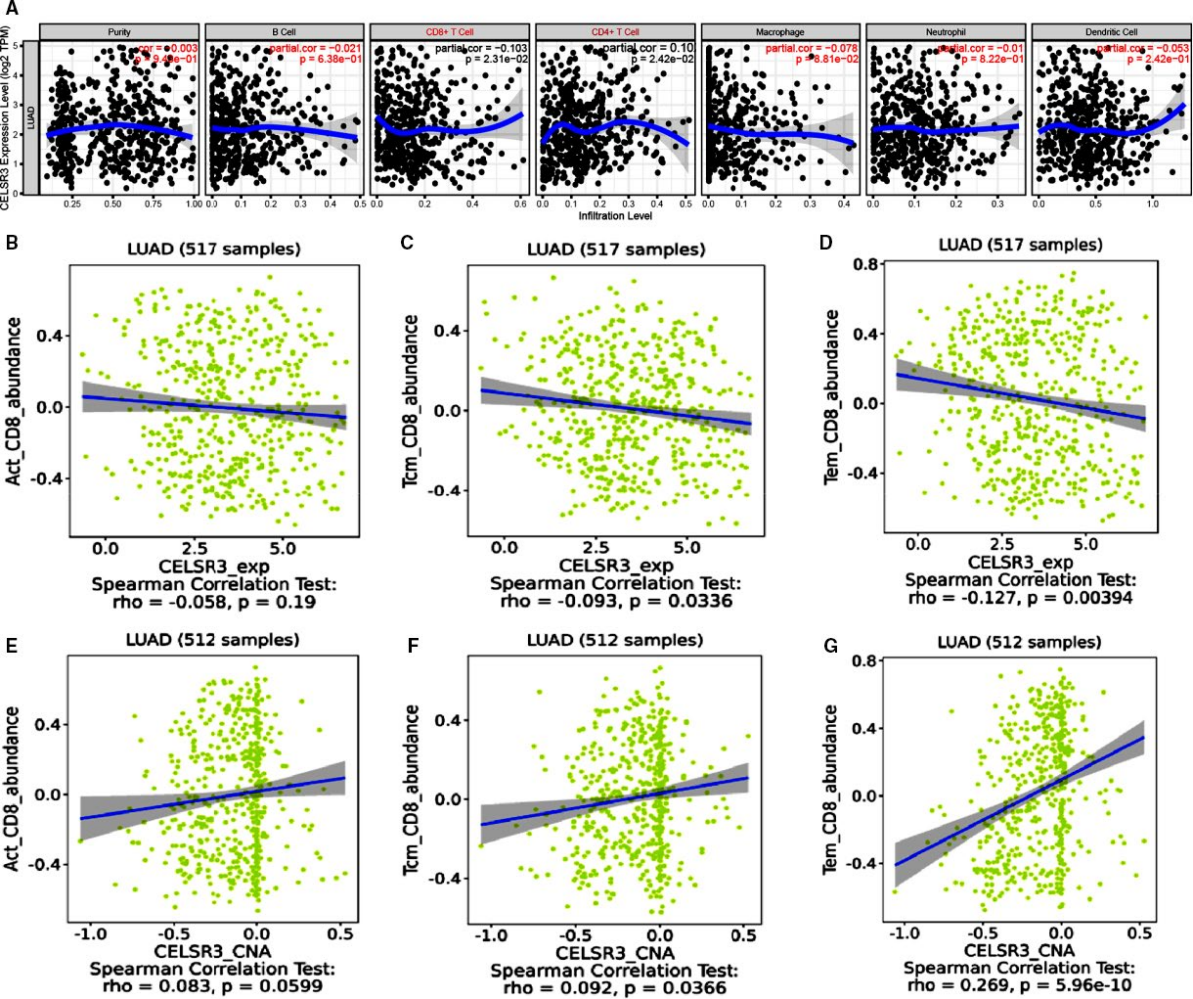
Correlation of *CELSR3* expression with infiltrating levels of CD8+ T cells. A, *CELSR3* expression has a significant correlation with immune infiltration level (TIMER database). B‐D, Relations between CD8+ T cells subtypes and expression of *CELSR3* (TISIDB database). E‐G, Relations between CD8+ T cells subtypes and copy number of *CELSR3* (TISIDB database)

We analysed the correlation between *CELSR3* expression and six types of immune cells infiltration (B cells, CD4+ T cells, CD8+ T cells, neutrophils, macrophages and dendritic cells). The study showed that *CELSR3* expression levels significantly correlated with the infiltrating levels of CD8+ T cells (***r* = **−**0.103, *P* = 2.31e**−**2**) and CD4+ T cells (***r* = 0.102, *P* = 2.42e**−**02**) in LUAD, but had no significant correlations with B cells (*r* = −0.021, *P* = 6.38e−01), macrophages (*r* = −0.078, *P* = 8.81e−02), neutrophils (*r* = −0.01, *P* = 8.22e−01) or dendritic cells (*r* = −0.053, *P* = 2.42e−01) (Figure 6A). The correlations between *CELSR3* expression and the CD8+T cells (Figure 6B‐G) and CD4+ T cells (Figure S2) were confirmed in the TISIDB database. The results showed that the expression levels of *CELSR3* were significantly negatively correlated with infiltration levels of Activated CD8+ T cells (*r* = −0.058, *P* = .19), Tcm CD8+ T cells (***r* = **−**0.093, *P* = .0336**) and Tem CD8+ T cells (***r* = **−**0.127, *P* = .00394**) (Figure 6B‐D). The results showed that the copy number levels of *CELSR3* were significantly positively correlated with the infiltration levels of Act CD8+ T cells (*r* = 0.083, *P* = .0599), Tcm CD8+ T cells (***r* = 0.092, *P* = .0336**) and Tem CD8+ T cells (***r* = 0.269, *P* = 5.9e**−**10**) (Figure 6E‐G).

### 3.7 Correlation of *CELSR3* expression with the CCL17/CCR4 axis

**FIGURE 7:**
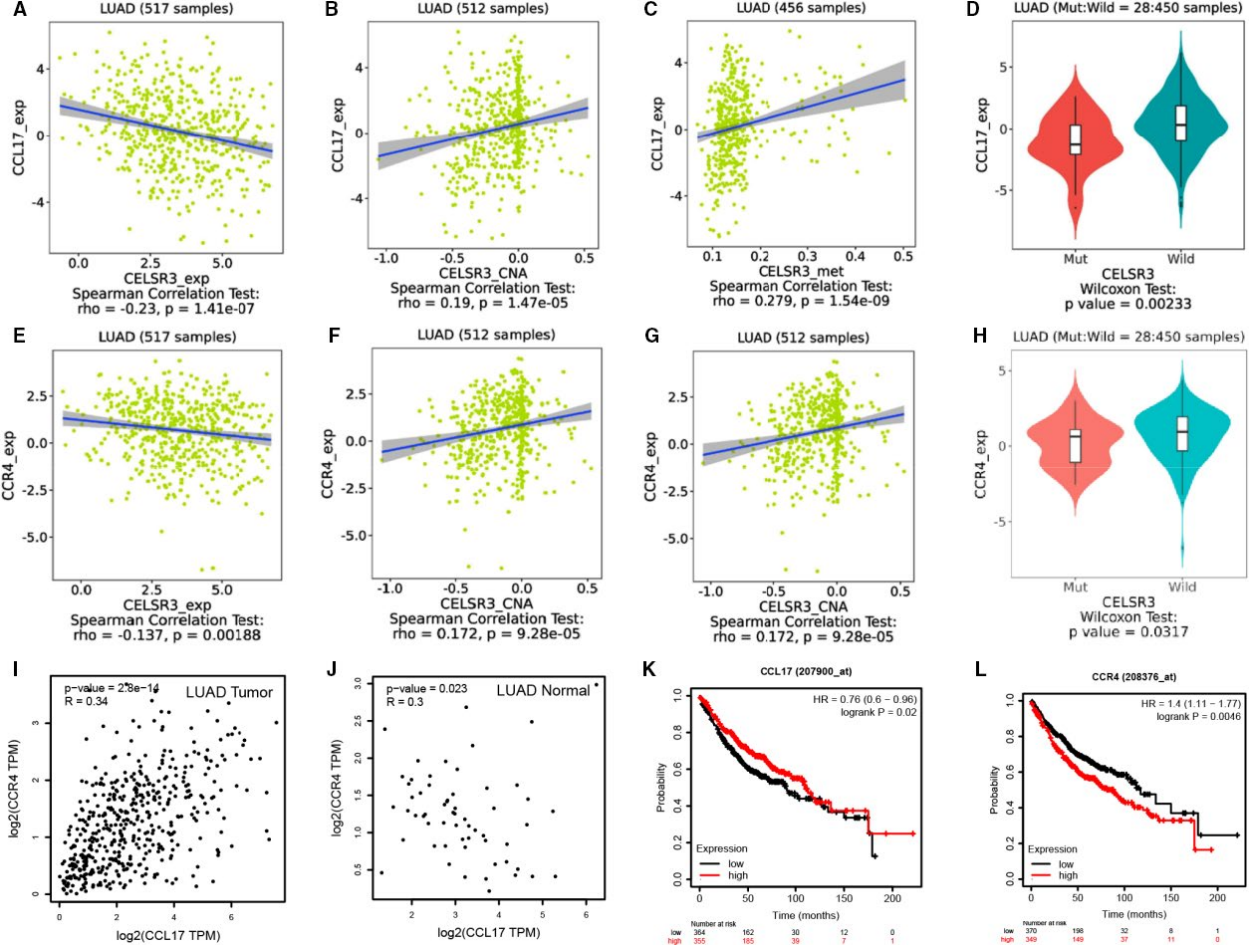
Correlation of *CELSR3* expression with levels of the CCL17/CCR4 axis. A‐D, Relations between CCL17 and expression, copy number, methylation and mutation of *CELSR3* in the TISIDB database. E‐H, Relations between CCR4 and expression, copy number, methylation and mutation of *CELSR3* in the TISIDB database. I, J, Correlation of CCL17 with CCR4 in LUAD tumour and normal (GEPIA database). K, L, CCL17 and CCR4 expressions are correlated with prognosis in LUAD (Kaplan‐Meier plotter database)

We examined the correlations between *CELSR3* expression and the CCL17/CCR4 axis in the TISIDB database. Relationships between CCL17 and expression (***r* = **−**0.23, *P* = 1.41e**−**07**), copy number (***r* = 0.19, *P* = 1.47e**−**05**), methylation (***r* = 0.279, *P* = 1.54e**−**09**) and mutation (***P* = .00233**) of *CELSR3* are shown in Figure 7A‐D. The relationships between CCR4 and expression (***r* = **−**0.137, *P* = .00188**), copy number (***r* = 0.172, *P* = 9.28e**−**05**), methylation (***r* = 0.269, *P* = 6.28e**−**09**) and mutation (***P* = .0317**) of *CELSR3* are shown in Figure 7E‐H. The relationship between *CCL17* and *CCR4* was retrieved in GEPIA, where it was shown that *CCR4* and *CCL17* expression is significantly correlated in LUAD tumour(***r* = 0.34, *P* = 2.8e**−**14**) and LUAD normal (***r* = 0.3, *P* = .023**) (Figure 7I‐J). Interestingly, high *CCL17* (***P* = .02**) and *CCR4* (***P* = .0046**) expressions have a significant correlation with prognosis in LUAD (Figure 7K‐L). The correlations between *CELSR3* expression and the chemokines (or receptors) need further study to fully validate these findings.

### 3.8 Correlation of markers of CD8+ T cells with the CCL17/CCR4 axis

To analyse the relationship between CD8+ T cells and the CCL17/CCR4 axis, we examined the correlations between markers of CD8+ T cells and the CCL17/CCR4 axis in the TIMER database. The relationships between CD8A and CCL17 expression (***r* = 0.156, *P* = 3.69e**−**04**) and CCR4 (***r* = 0.539, *P* = 3.37e**−**40**) are shown in Table 3 (Figure S3A,B). The relationships between CD8B and the expression of CCL17 (***r* = 0.13, *P* = 3.04e**−**03**) and CCR4 (***r* = 0.428, *P* = 2.09e**−**24**) are shown in Table 3 (Figure S3C,D). There is a positive and statistically significant (*P* < .05) correlation between CD8+ T cells and the CCL17/CCR4 axis.

**TABLE 3:** Correlation of markers of CD8+ T cells with the CCL17/CCR4 axis (TIMER database)

	CCL17		CCR4	
	cor	*P*‐value	cor	*P*‐value
CD8A	0.156	3.69E−04	0.539	3.37E−40
CD8B	0.13	3.04E−03	0.428	2.09‐24

## 4 DISCUSSION

Lung adenocarcinoma severely affects human health and is associated with significant morbidity and mortality. Recently, a relationship between the expression of the *CELSR* family and tumours, such as adult brain tumours ^25^ and ovarian cancer,^26^ actually has also been reported. Although some members of the CELSR family have been shown to play a key role in cancer, the exact roles of *CELSR3* in LUAD remained unclear. In this study, we analysed the expression and value of *CELSR3* in LUAD. In addition, we also investigated the association between *CELSR3* expression and immune infiltration, as well as the molecular mechanism leading to different levels of immune infiltration.

Our findings have shown *CELSR3* expression to be significantly higher in LUAD tissues than in adjacent normal tissues. To the best of our knowledge, this is the first report of a consistent relationship between elevated *CELSR3* mRNA levels and poor prognosis and early tumour progression in patients with LUAD; although other studies have described the role of *CELSR3* in the occurrence and development of several tumour types, including liver^27^ and oral squamous carcinomas.^10^ These findings indicate that *CELSR3* may play an oncogenic role and be an important prognostic indicator in LUAD.

To further explore the functions and mechanism of *CELSR3* in LUAD, we finally performed GSEA using the target gene sets to help identify the networks of target kinases, miRNAs and transcription factors. *CELSR3*, as the key crucial signalling molecule in the WNT/PCP pathway, is thought to be involved in tumorigenesis and metastasis.^28^ The main conclusion we draw from our findings is that the functional network of *CELSR3* is predominantly involved in the cell adhesion molecules, cell cycle G2/M‐phase transition, the spliceosome, DNA replication and RNA transport pathways. The practical network of *CELSR3* transcription plays a role in the structural constituents of ribosomes, gene expression and the cell cycle. The proliferation, apoptosis, invasion and metastasis of tumours are the result of synergy of several multi‐signalling pathways, including those of cell adhesion molecules,^29^ cell cycle G2/M‐phase transition,^30^ the spliceosome,^31^ DNA replication^32^ and as structural constituents of ribosomes.^33^ Furthermore, *CELSR3*‐related functional classification and the KEGG pathway are closely related to tumour development, invasion and metastasis. This shows the importance of elucidating the functional mechanism underlying *CELSR3* expression.

To understand the mechanisms of how *CELSR3* regulate tumour development, invasion and metastasis in LUAD, we examined the associated transcription factors and kinases. Our findings revealed that in LUAD, *CELSR3* was associated with a network of kinases, including PLK1, ATR, CHEK1, CHEK2, CDK1 and CDK2. The kinase CDK1,^34^ a member of the family of serine‐threonine kinases, and CDK2,^35^ a cyclin‐dependent kinase, regulate mitosis and the cell cycle. In fact, ATR is one of the core kinase regulators for genomic stability. It can initiate genomic instability repair and regulate cell responses,^36^ and its inhibitors can kill tumour cells and act synergizes with chemoradiotherapy.^37^ The Chek1/2 inhibitor regulates G2/M cell cycle arrest, and this Chek1/2 combination increases micronucleus formation in vitro. In LUAD, *CELSR3* may regulate DNA replication, repair and cell cycle progression,^38^ which is consistent with our findings. In 2011, Hanahan described 10 signature features of tumours, of which ‘continuous proliferation’ was the most important.^39^ It is generally accepted that the primary cause of this continuous proliferation is the abnormal expression of cell cycle‐associated proteins in proliferation, differentiation, apoptosis, multiplication and tumour development. E2F family is one of the key links in the cell cycle and proliferation regulation networks,^40^ and abnormal expression of E2F1^41^ and E2F4^41^ regulated common target genes contributes to cancer development and progression. Research has revealed an association between elevated E2F1 and E2F4 expressions in patients with LUAD and poor disease prognosis. In summary, kinases and transcription factors related to *CELSR3* are closely related to the proliferation, cell cycle, invasion and metastasis of tumour cell.

We have further verified a potential role of *CELSR3* in LUAD through interfering with the *CELSR3* expression. Our findings have revelled that *CELSR3* down‐regulation significantly suppressed cell proliferation and markedly suppressed tumour growth in vitro. In addition, *CELSR3* knockdown dramatically reduced the migration and invasion abilities of LUAD cells in vitro. These findings agree with previous analysis showing that *CELSR3* can affect cell growth, invasion and metastasis in LUAD. Collectively, this experiment indicated that down‐regulation of *CELSR3* might suppress cell proliferation, migration and invasion by regulating the cell cycle signalling pathway in LUAD cells.

We have also demonstrated the role of *CELSR3* in LUAD immune infiltration and analysed the correlation between *CELSR3* and different levels of infiltration of immune cells. Analysis of public database revealed a negative correlation between the level of CD8+ T cells and expression of *CELSR3* mRNA. Our results showed that the *CELSR3* expression level was negatively associated with infiltration levels of CD8+ T cells subtypes, including Act CD8+ T cells, Tcm CD8+ T cells, and Tem CD8+ T cells. As an important part of adaptive immunity, CD8+T cells play a key role in the clearance of various pathogenic microorganisms and tumour cells. Most cancers, including LUAD, promote cancer progression by inhibiting T cell function and overexpressing inhibitory ligands to evade immune responses. As far as we know, the proliferation and activation of CD8+T lymphocytes directly kill tumour cells and have anti‐tumour effect.^42^ Indeed, successful delivery of CD8+ effector T cells across the tumour vessels is considered to be a key determinant of anti‐tumour immunity.^43^ Although CD8+ cytotoxic T lymphocytes (CTLs) play an important role in tumour regression, CD4+ cells are necessary for CTLs to enter tumour tissue.^44^ Furthermore, our results indicated a certain direction for *CELSR3* in the level of immune cell infiltration. In summary, our results showed that *CELSR3* plays an important role in regulating CD8+T cells infiltration in LUAD.

Numerous studies have shown that the interaction between chemokines and cell receptors allows targeted function of circulating immune cells. Different types of immune cell infiltration are closely regulated by chemokines that regulate tumour immunity and biological phenotypes of tumours, in addition to influencing tumour progression, therapy and prognosis.^45^ To elucidate effector T cell differentiation and protective immunity, we studied the chemokines and receptors associated with *CELSR3* in the TISIDB database and performed further analysis using the GEPIA, KM plotter, and TIMER databases (Table S1‐S3). Interestingly, we found that CCL17/CCR4 was the only axis associated with *CELSR3* expression that was statistically significant. Using the TISIDB database, we studied relationships between the CCL17/CCR4 axis and *CELSR3* expression, copy number, methylation and mutation. Our findings revealed negative correlation between levels of expression of the CCL17/CCR4 axis and *CELSR3* expression; this trend was reversed when *CELSR3* was either methylated or mutated. This is the first time, as far as we know, that *CELSR3* influences the infiltration of CD8+T cells through the CCL17/CCR4 axis. The CCL17/CCR4 axis is known to basically regulate gene expression and play an important role in T cell differentiation and function, which is fairly significant. Previous studies have shown that the CCL17/CCR4 receptor axis represents a group of potential new targets for the treatment and prevention of autoimmune diseases of the central nervous system.^46^ Indeed, DC‐derived CCL17 was found to promote the interaction between DCs and CD8+ T cells, thus activating CD8+ T cells.^47^, ^48^ We have found that CD8+ T cells to be significantly and positively associated with the CCL17/CCR axis (*P* < .05). These findings are in accordance with those of our previous reports. Moreover, the CCL17/CCR4 axis may play an essential role in the differentiation and function of CD8+ T cells.

This study has several important limitations. First, as it has been based on data retrieved from published articles and public repositories, the quality of the data can influence the study outcomes. In *in* vitro experiments, we only verified the function of proliferation, invasion and metastasis. We will further verify the results of the immunocorrelation analysis in in vivo experiments. Second, the number of samples in the database is continuously monitored and expanded, which may affect the results of our study. Consequently, we aim to collect more clinical cases to verify the prognostic value of *CELSR3* in LUAD. Third, the accuracy and selection of the statistical methods used by the database to analyse the data may influence the interpretation of the research results. More effective analytical means and methods need to be learned and developed. However, we obtained similar results from analysing multiple databases, thus supporting our research conclusions. In future analyses, additional basic and clinical trials will be necessary to verify the mechanism of action and prognostic value of *CELSR3* in LUAD.

## 5 CONCLUSIONS

To the best of our knowledge, this is the first study to demonstrate high specific expression of *CELSR3* mRNA and its prognostic effects in LUAD. Down‐regulation of *CELSR3* significantly suppressed cell proliferation and dramatically reduced the ability of of LUAD cells to migrate and invade. More importantly, this is the first study to show that *CELSR3* may influence the level of infiltration level of CD8+ T cells through the CCL17/CCR4 axis in LUAD. Obviously, in future analyses, more basic and clinical trials will be required to verify diagnostic and immunotherapeutic value of *CELSR3* in LUAD.

## CONFLICT OF INTEREST

The authors declare that there is no conflict of interests. No animal or human studies were carried out by the authors for this article.

## AUTHOR CONTRIBUTIONS

**Yishuai Li:** Conceptualization (equal); data curation (lead); formal analysis (lead); funding acquisition (supporting); investigation (equal); methodology (equal); project administration (equal); resources (equal); software (equal); supervision (equal); validation (equal); Visualization (lead); writing‐original draft (lead); writing‐review and editing (equal). **Longyu Zhu:** Data curation (equal); validation (equal); visualization (equal); writing‐original draft (equal); writing‐review and editing (supporting). **Ran Hao:** Data curation (equal); methodology (equal); validation (equal); visualization (equal); writing‐original draft (supporting). **Yuejun Li:** Conceptualization (equal); data curation (equal); formal analysis (equal); investigation (equal); methodology (equal); project administration (equal). **Qinfei Zhao:** Data curation (equal); formal analysis (equal); methodology (equal); project administration (equal); writing‐original draft (supporting). **Shujun Li:** Conceptualization (lead); data curation (equal); formal analysis (equal); funding acquisition (lead); investigation (lead); methodology (equal); project administration (lead); resources (equal); supervision (lead); validation (equal); visualization (equal); writing‐original draft (equal); writing‐review and editing (lead).

## Supporting information

Supplementary Material
