# Supplementary material for: Systematic expression analysis of the CELSR family reveals the importance of CELSR3 in human lung adenocarcinoma
Source: J Cell Mol Med. 2021 Apr 3;25(9):4349–62. doi: 10.1111/jcmm.16497 (PMC8093986; doi:10.1111/jcmm.16497)

Supplementary Table 1

| **Relations between chemokines and expression of *CELSR3*(TISIDB), CD8A and CB8B(TIMER), chemokines prognosis(Kaplan-Meier plot) in LUAD.** | | | | | | | | |
| --- | --- | --- | --- | --- | --- | --- | --- | --- |
| Description | spearman correlation Test rho | *p-*value | OS | | CD8A | | CD8B | |
|  |  |  | HR | *p*-value | cor | *p*-value | cor | *p*-value |
| CCL2 | -0.093 | **0.0351** | 1.78(1.4-2.27) | **1.96E-06** | 0.269 | ***6.39E--10*** | 0.238 | ***4.78E-08*** |
| CCL3 | -0.011 | 0.799 |  |  |  |  |  |  |
| CCL4 | 0.006 | 0.885 |  |  |  |  |  |  |
| CCL5 | -0.063 | 0.152 |  |  |  |  |  |  |
| CCL7 | 0.047 | 0.281 |  |  |  |  |  |  |
| CCL8 | 0.033 | 0.46 |  |  |  |  |  |  |
| CCL11 | 0.065 | 0.141 |  |  |  |  |  |  |
| CCL13 | -0.195 | **8.13E-06** | 1.08(0.86-1.37) | 0.5 |  |  |  |  |
| CCL14 | -0.322 | **7.63E-14** | 0.97(0.77-1.23) | 0.81 |  |  |  |  |
| CCL15 | -0.211 | **1.30E-06** | 0.97(0.77-1.23) | 0.81 |  |  |  |  |
| CCL16 | -0.23 | **1.41E-07** | 0.89(0.7-1.130 | 0.33 |  |  |  |  |
| CCL17 | -0.23 | **1.41E-07** | 0.76(0.6-0.96) | **0.02** | 0.156 | ***3.69E-04*** | 0.13 | ***3.04E-03*** |
| CCL18 | -0.106 | **1.58E-02** | 1.46(1.15-1.85) | **0.0016** | 0.408 | ***4.26E-07*** | 0.321 | ***8.33E-14*** |
| CCL19 | -0.162 | **2.15E-03** | 1.16(0.92-1.47) | 0.2 |  |  |  |  |
| CCL20 | 0.029 | 5.08E-01 |  |  |  |  |  |  |
| CCL21 | -0.099 | **2.45E-02** | 1.87(1.47-2.38) | **1.90E-07** | 0.454 | 0 | 0.394 | 0 |
| CCL22 | -0.168 | **1.22E-04** | 0.69(0.54-0.87) | **0.0018** | 0.229 | ***1.49E-07*** | 0.12 | ***6.46E-03*** |
| CCL23 | -0.248 | **1.22E-08** | 0.83(0.66-1.05) | 0.12 |  |  |  |  |
| CCL24 | -0.074 | 9.22E-02 |  |  |  |  |  |  |
| CCL26 | 0.098 | **2.54E-02** | 1.57(1.23-2) | **0.00028** | 0.227 | ***1.85E-07*** | 0.228 | ***1.74E-07*** |
| CCL28 | -0.058 | 1.88E-01 |  |  |  |  |  |  |
| CX3CL1 | -0.075 | 8.77E-02 |  |  |  |  |  |  |
| CXCL1 | -0.026 | 5.57E-01 |  |  |  |  |  |  |
| CXCL2 | -0.036 | 4.08E-01 |  |  |  |  |  |  |
| CXCL3 | 0.003 | 9.40E-01 |  |  |  |  |  |  |
| CXCL5 | -0.049 | 2.66E-01 |  |  |  |  |  |  |
| CXCL6 | -0.084 | 5.71E-02 |  |  |  |  |  |  |
| CXCL8 | 0.038 | 3.82E-01 |  |  |  |  |  |  |
| CXCL9 | 0.052 | 2.42E-01 |  |  |  |  |  |  |
| CXCL10 | 0.045 | 3.09E-01 |  |  |  |  |  |  |
| CXCL11 | -0.01 | 8.15E-01 |  |  |  |  |  |  |
| CXCL12 | -0.122 | **1.06E-02** | 0.63(0.5-0.8) | **0.00011** | 0.371 | ***0*** | 0.33 | ***2.21E-14*** |
| CXCL13 | -0.065 | 1.39E-01 |  |  |  |  |  |  |
| CXCL14 | -0.103 | **1.96E-02** | 0.72(0.56-0.91) | **0.0069** | 0.132 | ***2.69E-03*** | 0.128 | ***3.63E-03*** |
| CXCL16 | -0.23 | **1.42E-07** | 0.69(0.54-0.89) | **0.0032** | 0.044 | 3.19E-01 | 0.003 | 0.941 |
| CXCL17 | -0.194 | **8.86E-06** | 0.91(0.72-1.16) | 0.46 |  |  |  |  |
| XCL1 | -0.05 | 2.57E-01 |  |  |  |  |  |  |
| XCL2 | -0.084 | 5.51E-02 |  |  |  |  |  |  |

| Supplementary Table 2  **Relations between receptors and expression of CELSR3(TISIDB), CD8A and CB8B(TIMER), receptors prognosis(Kaplan-Meier plot) in LUAD.** | | | | | | | | |
| --- | --- | --- | --- | --- | --- | --- | --- | --- |
| Description | spearman correlation Test rho | *p-*value | OS | | CD8A | | CD8B | |
|  |  |  | HR | *p*-value | cor | *p*-value | cor | *p*-value |
| CCR1 | -0.092 | ***0.0373*** | 1.18(0.93-1.49) | 0.17 |  |  |  |  |
| CCR2 | -0.21 | ***1.57E-06*** | 1.37(1.08-1.73) | ***0.0081*** | 0.579 | **0** | 0.489 | **0** |
| CCR3 | -0.058 | 0.185 |  |  |  |  |  |  |
| CCR4 | -0.137 | ***0.00188*** | 1.4(1.11-1.77) | ***0.0046*** | 0.539 | ***3.37E-40*** | 0.428 | ***2.09E-24*** |
| CCR5 | -0.053 | 0.232 |  |  |  |  |  |  |
| CCR6 | -0.18 | ***4.06E-05*** | 0.79(0.62-1) | ***0.0454*** | 0.394 | ***0*** | 0.292 | ***1.62E-11*** |
| CCR7 | -0.133 | ***2.50E-03*** | 0.92(0.73-1.16) | 0.46 |  |  |  |  |
| CCR8 | -0.055 | 0.213 |  |  |  |  |  |  |
| CXCR1 | -0.141 | ***1.29E-03*** | 0.98(0.77-1.24) | 0.88 |  |  |  |  |
| CXCR2 | -0.243 | ***2.55E-08*** | 1.01(0.8-1.27) | 0.96 |  |  |  |  |
| CXCR4 | -0.138 | ***1.65E-03*** | 0.62(0.49-0.79) | ***8.00E-05*** | 0.564 | ***0*** | 0.479 | ***0*** |
| CXCR5 | -0.049 | 2.68E-01 |  |  |  |  |  |  |
| CXCR6 | -0.087 | ***4.89E-02*** | 0.67(0.53-0.85) | ***0.00077*** | 0.888 | 0 | 0.77 | 0 |
| CX3CR1 | -0.202 | ***4.02E-06*** | 0.48(0.38-0.61) | ***1.60E-06*** | 0.091 | ***3.83E-02*** | 0.024 | 5.88E-01 |

| Supplementary Table 3  **Relations between chemokine/receptor axis in LUAD Tumor and Normal(GEPIA).** | | | | |
| --- | --- | --- | --- | --- |
| Description | Tumor | | Normal | |
|  | cor | *p*-value | cor | *p*-value |
| CCL2/CCR2 | 0.3 | 7.60E-12 | -0.21 | 0.11 |
| CXCL12/CXCR4 | 0.38 | 0 | 0.14 | 0.29 |
| CCL17/CCR4 | 0.34 | ***2.80E-14*** | 0.3 | ***0.023*** |

Supplementary Table 4

**The siRNA sequences used in cell transfection**

| Name | Sequences(5'to3') |
| --- | --- |
| CELSR3 siRNA#1 | GGAUGUGGCCAUGUUCCAUTT |
| CELSR3 siRNA#2 | GCAAUGACCUCCUGUCCUATT |
| CELSR3 siRNA#3 | CCUCCAUCCUUGCCUCUUUTT |
| si-con | UUCUCCGAACGUGUCACGUTT |

Supplementary Table 5

**The primers used in qRT-PCR**

| Name | Sequences(5'to3') |
| --- | --- |
| CELSR3 forward | AGAGTATGCCTTGCGCATCA |
| CELSR3 reverse | ACAGAAACTTGGAAGGGCGT |
| GAPDH forward | ACACCCACTCCTCCACCTTT |
| GAPDH reverse | TTACTCCTTGGAGGCCATGT |

Supplementary Figure 1 Transcription factors/kinaases prognosis in LUAD(K-M plot) (A-I) and the relationship between CELSR3 and transcription factors/kinaases(GEPIA database) (J).


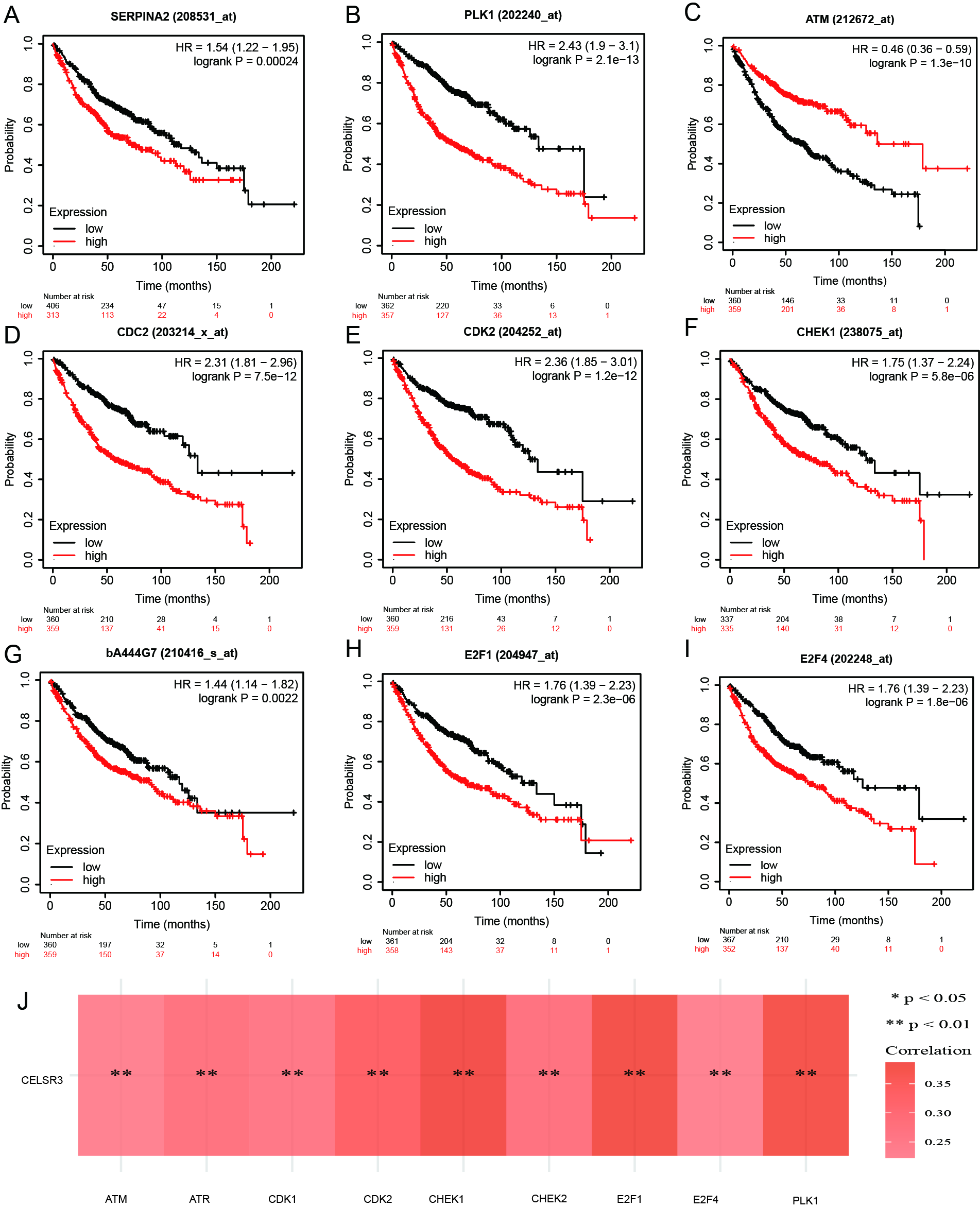


Supplementary Figure 2 Correlation of CELSR3 expression with infiltrating levels of CD4+ T cells. (A) CELSR3 expression has a significant correlation with immune infiltration level (TIMER database). (B-D) Relations between CD4+ T cells and expression of CELSR3 (TISIDB database). (E-G) Relations between CD4+ T cells and copy number of CELSR3 (TISIDB database).


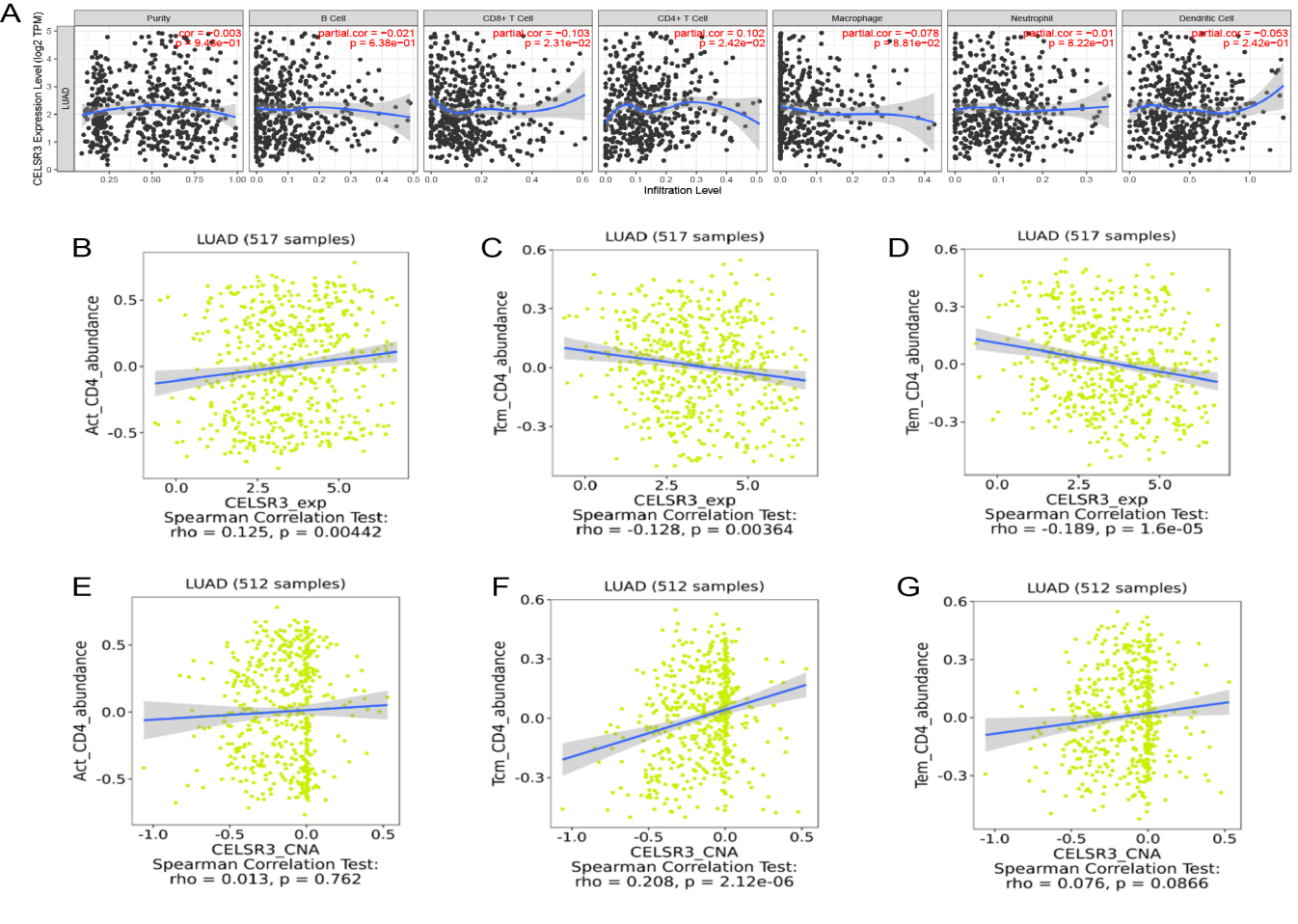


Supplementary FIGURE 3 Correlation of markers of CD8+T cells with the CCL17/CCR4 axis (TIMER database).

.
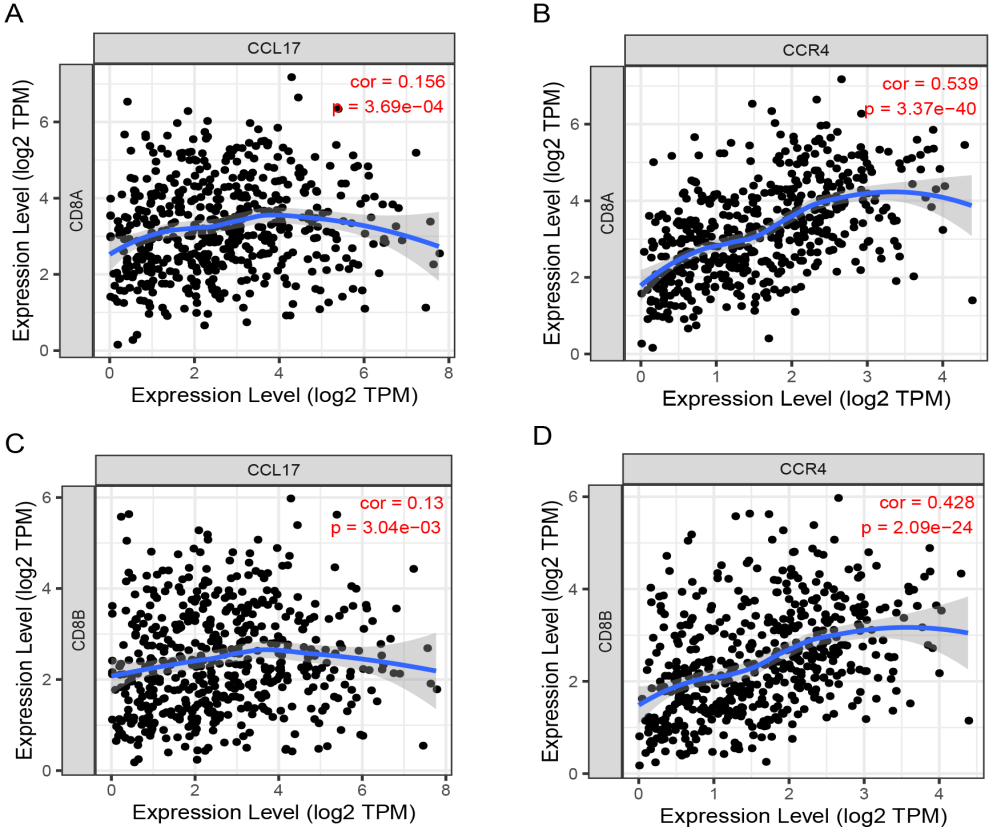


Supplementary FIGURE 4 si-CELSR3 ability of silencing.


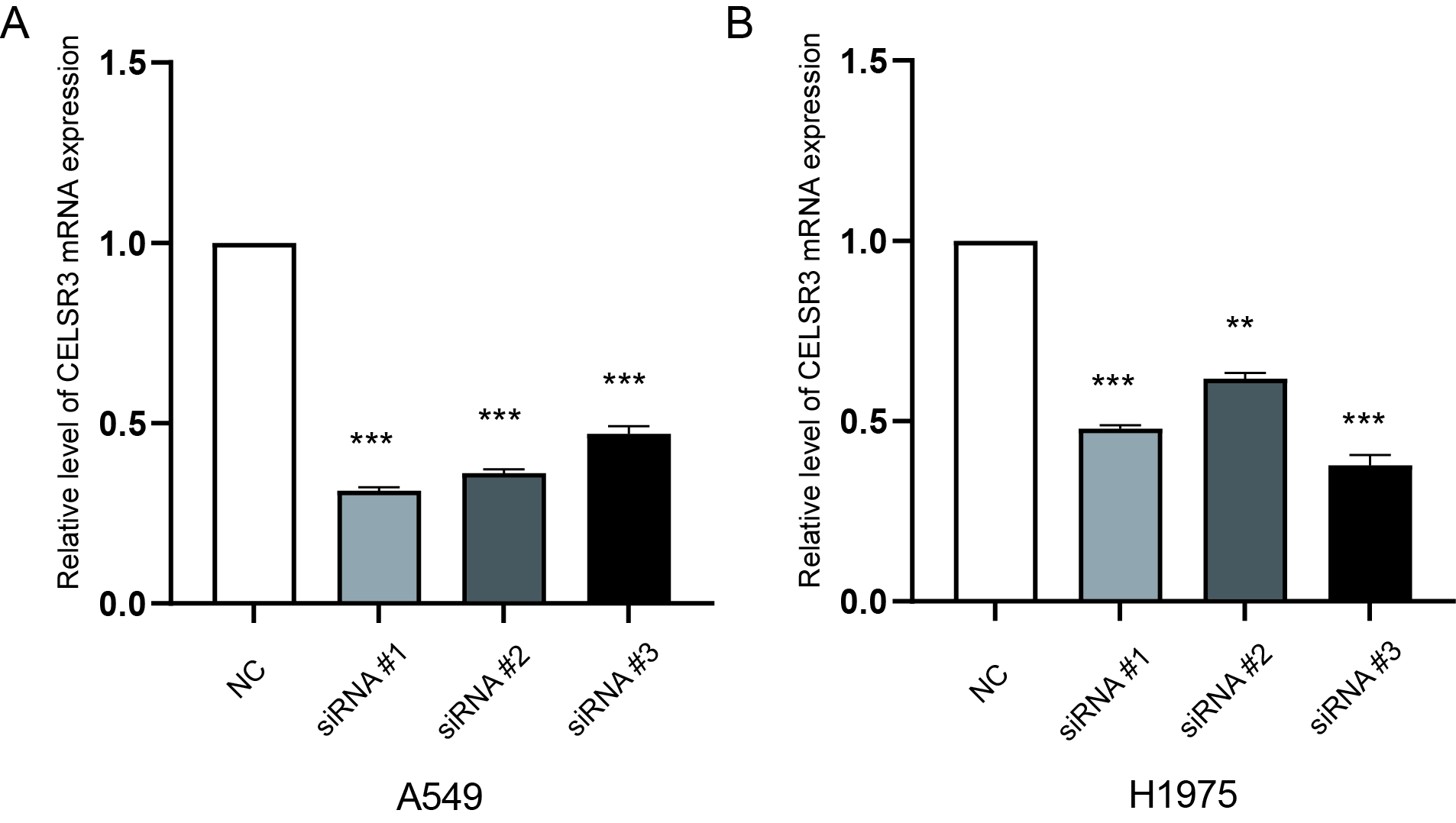

Supplement: Supplementary file 1 — Supplementary Material [file JCMM-25-4349-s001.docx]
